# Enteral Feeds: Benefits and Drawbacks Associated with Blenderized Feeds and Commercial Formulas—A Narrative Review

**DOI:** 10.3390/medicina62040745

**Published:** 2026-04-13

**Authors:** Athanasios Migdanis, Ioannis Migdanis, Souzana K. Papadopoulou, Constantinos Giaginis, Maria Anna Polyzou Konsta, Andreas Kapsoritakis

**Affiliations:** 1 Nutrition and Dietetics Department, University General Hospital of Larissa, Viopolis Mezourlo, P.C. 41110 Larissa, Greece; 2 Nutrition and Dietetics Department, University of Thessaly, Argonafton 1C, P.C. 42132 Trikala, Greece; 3 Oncology Clinic and Hemodialysis Unit, “E. Patsidis” Health Group, Department of Clinical Nutrition, Theofrastou 75, P.C. 41335 Larissa, Greece; i.migdanis@aegeancollege.gr; 4 Faculty of Medicine, University of Thessaly, Viopolis Mezourlo, P.C. 41110 Larissa, Greece; kapsoritakis@uth.gr; 5 MSc Program Nutrition in Health and Disease, Faculty of Medicine, University of Thessaly, Viopolis Mezourlo, P.C. 41110 Larissa, Greece; 6 Department of Nutritional Sciences and Dietetics, International Hellenic University, Nea Moudania, P.C. 57001 Thessaloniki, Greece; sousana@the.ihu.gr; 7 Department of Food Science and Nutrition, University of the Aegean, Myrina, P.C. 81400 Lemnos, Greece; cgiaginis@aegean.gr; 8 Department of Nephrology, General Hospital of Larissa, Tsakalof Athanasiou 1, P.C. 41221 Larissa, Greece; polyzoukonsta@yahoo.com; 9 Department of Gastroenterology, University General Hospital of Larissa, Viopolis Mezourlo, P.C. 41110 Larissa, Greece

**Keywords:** blenderized tube feeds, commercial enteral formulas, nutritional status, anthropometric indices, food allergies, gastrointestinal tolerance, microbial content, gastrointestinal microbiota, tube clogging, nutritional composition, financial cost

## Abstract

Enteral feeds can be classified as two major types: commercial enteral formulas and blenderized enteral feeds. When deciding the most appropriate type of feed for a patient, several parameters need to be taken into consideration. Benefits associated with the use of blenderized tube feeds include giving a sense of normalcy to patients, having a positive impact on anthropometric indices, allowing for careful control over known food allergens, being associated with improved gastrointestinal tolerance and improved gut microbial composition (mainly in pediatric populations) and being generally less costly compared with commercial formulas. On the other hand, commercial enteral formulas have the advantages of being generally sterile, less likely to cause tube clogging and having a known and consistent nutrition composition. Further studies, specifically well-designed randomized controlled trials including adult populations, that will emphasize the impact of blenderized enteral feeds on various clinical outcomes are warranted. Clinicians need to be conscientious and educated regarding safe food practices for blended food preparation, as well as the characteristics of commercial formula options available to help assist patients in selecting the proper feed for their nutritional needs and clinical condition.

## 1. Introduction

**Figure 1:**
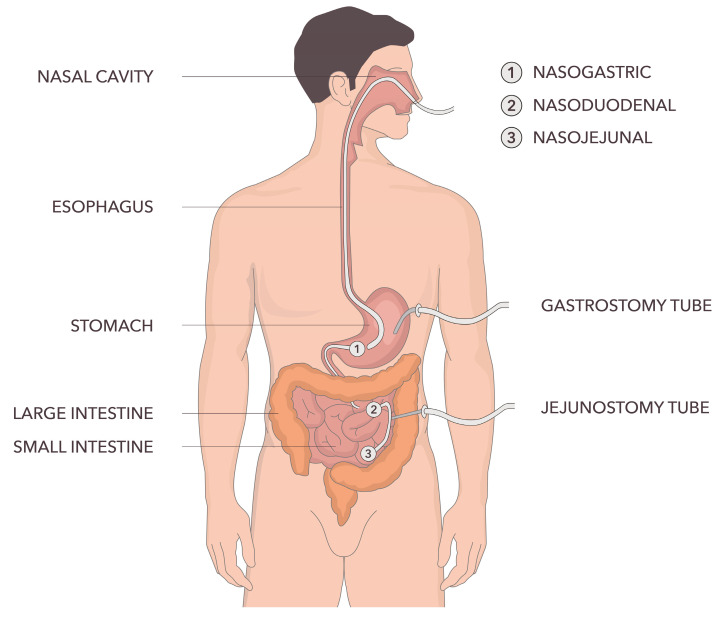
Types of enteral tubes.

Enteral nutrition (EN) refers to different forms of nutritional support including oral nutritional supplements as well as tube feeding via nasogastric, nasoenteral or percutaneous tubes [1], as outlined in Figure 1. EN is given to patients whose oral intake is inadequate or those who cannot eat normal food as consumed at home or as offered by the catering system of a hospital [2]. Inadequate intake can be caused by a variety of medical or physiological reasons. Some symptoms related to diseases or their treatments such as nausea, taste changes or poor appetite can affect oral intake. Neurological conditions such as motor neuron disease or Parkinson’s disease, as well as dysfunction of the swallowing mechanism as a result of conditions like head and neck cancers or stroke, can often cause dysphagia, which can also affect oral intake [3]. Other patients who often cannot meet their nutritional requirements and might also need artificial nutrition support are those in a diminished state of consciousness or with a markedly changed mental status as a result of respiratory problems, hepatic encephalopathy, dementia or other conditions [4].

Impaired nutritional intake has also been described in patients with gastrointestinal (GI) problems such as short bowel syndrome (SBS) after surgical resections of the bowel, where pathophysiological and absorptive consequences depend on the extent and site of the bowel resection. According to the literature, EN in patients with SBS can help improve postsurgical intestinal adaptation, since after a small bowel resection, the residual gut alters in function and structure (characterized by cellular hyperplasia, villous hypertrophy and alteration of motility), which improves nutrient absorption [5,6].

Other (GI) disorders that might benefit from EN includes inflammatory bowel disease (IBD), which includes Crohn’s disease (CD) and ulcerative colitis (UC), and pancreatitis. There is evidence that exclusive enteral nutrition (EEN) can be very promising in the management of CD, as it is connected with induced remission, and in some cases, EEN has had comparable efficacy to steroid treatment in inducing remission in children and adults with CD but with significantly fewer side effects [7,8,9]. The exact mechanisms underlying the association between exclusive EEN and CD yet remain unclear, although there is evidence that it could modify the composition of the intestinal microbiome, which is essential in the pathogenesis of CD [10]. As far as pancreatitis is concerned, in patients with acute severe pancreatitis, early EN is associated with lower mortality and a decreased risk of infection. EN is found to have a beneficial effect on the structure and function of the intestinal mucosa, luminal nutrients and immune function [11,12].

EN is also indicated for patients who are malnourished or at risk of malnutrition and cannot meet their nutritional requirements orally. Specifically, for hospitalized patients, disease-related malnutrition is described as a complex syndrome resulting from an inadequate intake of nutrients and from a disease-related systemic inflammatory response [13]. Malnutrition can lead to an altered body composition, which can have a negative effect on mental and physical function [2]. It is also worth mentioning that there is a strong correlation between malnutrition and an increased risk of adverse clinical outcomes, which include functional decline, prolonged hospital stays and higher rates of morbidity and mortality [14]. According to the literature, up to one-third of hospital patients have malnutrition or are at risk of it at the time of admission [15,16]. Furthermore, a patient’s nutritional state frequently declines during their hospital stay [17]. Unfortunately, in a hospital setting, adequate nutrition is not often prioritized and medical treatment comes first for medical professionals.

The main objective of this review is to provide a comprehensive overview and discussion of the benefits and drawbacks of blenderized feeds and commercial formulas, regarding the practical, financial, clinical and psychological parameters that are of concern to clinicians, enterally fed patients and their caregivers.

### Types of Enteral Feeds

Enteral feeds can be classified into two major types: commercial enteral formulas and blenderized enteral feeds. Commercial enteral formulas are liquid nutritional mixtures providing nutrients from various sources like milk/soy proteins, maltodextrin/corn syrup carbohydrates and vegetable oils such as canola or soybean oil, all formulated for tube feeding [3]. Commercial enteral formulas can be separated into standard (polymeric), semi-elemental/elemental, immune-modulating and disease-specific.

Blended diet is a term used to describe the process of giving liquidized or blended food through the enteral feeding device of patients who are unable to meet their nutritional needs orally, via a tube, catheter or stoma to the GI tract. Such formulas consist of natural, whole-food ingredients that can be either commercially prepared or non-commercially prepared (i.e., homemade). Blenderized formulas can be used either at hospital or at home and have benefits as well as disadvantages.

Food-based blenderized feeds have increased in popularity in recent years since, generally, a segment of the population is shirking away from processed foods. Increased real food in enteral feeds is shown by the number of commercially available food-based formulas entering the market. According to evidence, enteral feeding was first used in ancient Egypt and Greece, where solutions made from milk, wheat and whey were infused as rectal enemas; hence, the concept of a blended diet is not new [18]. Rectal feeding was the artificial feeding method of choice for thousands of years due to the difficulty of accessing the upper gastrointestinal (GI) tract [19].

## 2. Methodology and Study Selection

A comprehensive literature search was performed using the ScienceDirect, PubMed, Ovid, Google Scholar, and Medline databases to identify all articles related to the subject under investigation published between January 1990 and December 2025. The literature search conducted was not restricted to specific languages. The search concentrated on scientific articles that examined the effect of blenderized and commercial enteral feeds on social, psychological, clinical and financial parameters. The following search terms were used: blenderized tube feeds, commercial enteral formulas, nutritional status, anthropometric indices, food allergies, gastrointestinal tolerance, microbial content, gastrointestinal microbiota, tube clogging, nutritional composition and financial cost. Inclusion criteria included articles on different types of enteral feeds and the advantages and disadvantages connected to them, studies on adult patients, studies on pediatric patients (<18 years of age), observational studies, clinical trials including randomized controlled trials, systematic reviews and meta-analyses, and narrative reviews. More specifically, studies that focused on assessing patient populations requiring enteral nutrition, were randomized into either receiving blenderized feeds or commercial formulas, and examined clinical or practical outcomes were prioritized. Moreover, studies that focused on enterally fed patient populations that transitioned from commercial formulas to a blended diet (cross-over design) were also of high priority. Only full-text articles published in peer-reviewed journals were considered. The exclusion criteria included studies conducted on animal models, studies about other types of artificial nutrition such as parenteral nutrition, case studies, expert opinions, letters to the editor, or conference abstracts without full-text publication. Given the narrative design, formal risk-of-bias tools were not systematically applied. However, emphasis was placed on high-quality evidence, prioritizing well-designed clinical studies, systematic reviews and meta-analyses. The articles included were reviewed and assessed in detail by two independent reviewers.

## 3. Benefits of a Blenderized Diet

### 3.1. Social and Psychological Implications

Blenderized feeds can have benefits as well as disadvantages. One of the benefits of food-based blended diets is the sense of normalcy it gives patients (adults or children) and caretakers from the use of regular foods. Patients and caretakers have an increased sense of control over the foods that are used, and this gives a positive sense of normality and de-medicalizes enteral feeding [20]. Blended diets help to normalize the tube experience, and benefits to the emotional wellbeing of the patient and psychosocial interactions of the whole family have been observed [20]. In a study from the United Kingdom, the authors aimed to explore the experiences of patients that were currently or had in the past used a blended diet via gastrostomy [20]. In terms of methodology, the data were collected via semi-structured interviews, in order to allow the participants to express their views. The results showed that a blended diet was associated with a sense of normality (compared with commercial feeds), according to the participants; furthermore, some participants made references to the social involvement associated with using home blended foods, as they felt like they were having the same food as the rest of the family. The authors concluded that both carers and patients reported a positive experience of a blended diet via gastrostomy, with the use of family food giving the feeling of normalizing enteral feeding. In a similar study by Soscia et al., the authors qualitatively assessed the parental experiences and perceptions of blenderized tube feeding for their children [21]. According to the results of the study, parents thought that blended feeding positively affected the experience of tube feeding and described the method as a mechanism to normalize artificial feeding and address some of the psychosocial implications of enteral tube feeding.

### 3.2. Anthropometric Indices

Common concerns when feeding artificially are meeting nutritional requirements, stabilizing weight and preventing impairment of nutritional status. It has been observed that a patient’s nutritional state often deteriorates during their hospital stay due to an illness-related loss of appetite, fasting for diagnostic tests, drug-related side-effects, diseases that impair the normal functioning of the digestive system and other reasons. In a study where researchers tried to explore the clinical outcomes of blended diets on gastrostomy-fed children, they observed improved growth trends, including weight z-score trends and statistically significant height z-scores, in children who transitioned from commercial enteral formulas (CEFs) to blenderized tube feeds (BTFs) [22]. Another study from the United States of America (USA) that was conducted on pediatric registered dietitians (RDs) yielded interesting results [23]. In this study, a survey instrument was distributed to members of the pediatric nutrition practice group to report their experiences of blenderized food by gastrostomy tube (BFGT) in clinical practice. The majority of the respondents reported positive outcomes with BFGT feeding, including reduced tube feeding intolerance, optimal growth and increased acceptance of oral feeding. In a similar study that was carried out on parents of tube-fed children, data were collected via an electronic survey [24]. The objective of the study was to explore parents’ reported experiences of CEFs and BTFs in their children. The sample was evenly represented by parents using CEFs (50.5%) and BTFs (49.5%). Parents reported fewer symptoms of tube feeding intolerance on BTFs, and their children more frequently met growth goals compared with formula feeding [24]. Moreover, a study by the Oley Foundation, a non-profit USA-based organization providing education and support to enterally and parenterally fed patients and care providers, observed that weight loss was actually significantly less likely to occur in patients using BTFs compared with those using commercial enteral formulas [25]. More specifically, 90% of pediatric patients and 85% of adult patients reported no weight loss when on BTFs, compared with 59% of children and 52% of adult participants reporting no weight loss when using commercial enteral formulas. At this point it is worth noting that some studies have shown that CEFs may be better than BTFs at maintaining weight and anthropometrics in certain patient populations [26,27]. This could be attributed to the significant variabilities in the calorie, macronutrient and some micronutrient compositions/contents of BTFs [28,29,30]. Nevertheless, research has suggested that when designed appropriately by a clinical dietitian, who must evaluate nutrient recommendations for age and medical condition to ensure adequacy, BTFs can actually meet nutrient requirements [31].

### 3.3. Food Allergies

Food-based BTFs may be an excellent approach for those with various food allergies or sensitivities, as they can avoid ingredients such as eggs, milk, soy and corn that are frequently found in commercially available enteral formulations [32]. BTFs can allow for careful control over known food allergens, as they can be excluded from the feed. It seems that patients and caregivers of those who are on home enteral nutrition generally positively view BTFs, because nutrition can be personalized based on dietary preferences and allergens can be avoided [33]. In a study of 433 caregivers of tube-fed children, participants completed an electronic survey to compare experiences of commercial formulas and blenderized tube feeding [24]. The results showed that one of the main reasons parents chose BTFs was to address food allergies and intolerances. The primary reasons parents did not use BTFs included a lack of knowledge or time constraints. Furthermore, in a study aiming to address enteral and parenteral applications of ketogenic diet therapy, the researchers showed that using a whole-food blenderized formula helped to avoid exposure to milk and soy protein allergens in the case of an epileptic infant [34]. The authors concluded that lactose intolerance and allergies to milk and soy proteins could be an existing concern in both adults and children and that in such cases a dairy and soy-free formula could be designed by blenderizing whole foods.

### 3.4. Improved Gastrointestinal Tolerance

Emerging scientific data suggest that BTFs are associated with improved gastrointestinal (GI) tolerance (Table 1 summarizes studies in the literature that concentrate on the effect of BTFs on gastrointestinal tolerance). A study from the US, which was conducted on children that had undergone Nissen fundoplication surgery, yielded interesting results [35]. In children with feeding disorders, Nissen fundoplication is often required to reduce the risk of aspiration or prevent gastroesophageal reflux disease (GERD). In such children, gastrostomy tube feeding is sometimes unavoidable and is associated with symptoms such as gagging and retching. In this specific study, the researchers found that after transitioning 33 post-fundoplication children from commercial formulas to a pureed diet by gastrostomy tube, 73% had a >50% reduction in gagging and retching. Moreover, 57% of the participating children made progress in advancing their oral intake. According to the authors of the study, it is unclear exactly how a pureed diet works to reduce retching and gagging. A possible explanation is that the higher viscosity of the feed causes the stomach to empty more slowly, which reduces dumping syndrome. It is also possible that compared with the response from formulas, pureed foods elicit a distinct hormonal response that favorably affects gastrointestinal mobility. In a 2018 study from Canada that was carried out on a chronically ill children population with swallowing difficulties, neurological impairment, and/or developmental delays being fed via a gastrostomy tube, results were comparable [31]. The participants were transitioned from commercial formula to blenderized feeds and were monitored for 6 months for changes in gastrointestinal symptoms, oral feeding and nutrient intake. The results of the study revealed that the percentage of patients vomiting > once a week significantly decreased from 76% to 53%, and the prevalence of gagging and/or retching also decreased considerably from 82% to 47%. In a 2020 Johns Hopkins study on children with neurological disorders being fed via a gastrostomy tube, the findings were also similar [22]. The children that participated in the study (n = 23) were switched from commercial formulas to either homemade blended diets, commercial blended diets or a combination of both. Three months after initiation of the blended diet, 95% of the patients experienced improvements in upper GI symptoms including retching, gagging, emesis, coughing and other concerning symptoms for dysphagia or aspiration. The authors of the study attributed the reduction in GI symptoms to the increased viscosity of blended diets, which resulted in a slower rate of gastric emptying. A possible assumption is that with whole foods, the digested chyme enters the small intestine at a rate that triggers a more regular hormonal response. As a result, it promotes more physiologic motility and lessens symptoms of emesis, diarrhea and retching. Additionally, elements of blended diets, such as complex polysaccharides, are digested more slowly and promote euglycemia. Interesting findings were also obtained from a randomized controlled study conducted in a rehabilitation center in Germany [36]. The study was carried out on 118 patients recovering from neurological damage (e.g., ischemic stroke), requiring enteral nutrition, who were randomized to receive either blenderized feed or commercial formula over a maximum of 30 days. The results showed that tube feeding with natural-based food was associated with a significant reduction in the number of watery stool evacuations and in diarrhea, compared with standard tube feeding. This reduction in watery defecations observed in the natural-based food-feed group could be related to the composition of the fiber mixture that differed between the two groups, in combination with other components from carrots (contained in the blenderized feed) such as pectins and acidic oligosaccharides, for which previous research has demonstrated beneficial effects [37,38]. In another prospective cohort study on children receiving blenderized diets versus conventional formula via feeding tube at the Boston’s children’s hospital, results interestingly showed less nausea, vomiting and abdominal pain in participants who were on blenderized diets [39]. Although, as documented thus far, there is research suggesting that blenderized formulas are associated with improved GI tolerance, future research should focus on large-scale randomized controlled trials, and in order to determine which formula results in better clinical outcomes, the formulas should be nutritionally equivalent in completely meeting the patient’s nutritional needs.

### 3.5. Gastrointestinal Microbiota

The relationship between gut microbiota and human health has been the subject of extensive research in recent years. There is significant evidence indicating an actual association between BD and increased intestinal bacterial diversity, which can have a positive impact on gut microbiota [31]. The BLEND study, a prospective study conducted on children being fed via a gastrostomy tube who were transitioned from commercial formulas to blenderized feeds, showed that BTFs were associated with increased bacterial diversity and species richness compared with commercial formulas [31]. In a prospective pilot study that tried to examine the effects of plant-based enteral nutrition on gut microbiota, results showed that plant-based enteral nutrition was well tolerated and improved the health of the microbiota in chronically ill children [40]. The children that participated in the study were on conventional enteral formula and were transitioned to plant-based EN for two months. The authors attributed the results to the higher amount of dietary fiber and other nutritionally dense ingredients of the plant-based EN. In another study that was carried out in Japan, the researchers aimed to evaluate the gut microbial communities in children receiving enteral nutrition with or without homemade BTFs [41]. The study concluded that homemade BTFs increased the gut microbial diversity and altered the gut microbial composition and networks towards a healthy state. Although understanding of the role of the microbiome in disease is still in very early stages, according to findings on the subject, several human diseases have been connected to declines in bacterial richness and diversity [42,43]. The variety of food components often included in BTFs such as dietary fiber and phytonutrients from natural fruit and vegetables and natural prebiotics from legumes could benefit the biodiversity of the microbiota [32,44,45]. The current literature on the effect of BTFs on the gut microbiome is unfortunately still limited, especially in adults, since the available research is mainly focused on pediatric populations. Future research should aim for large scale randomized controlled trials (RCTs) comparing the impact of BTFs and CEFs on outcomes such as gut microbiome changes.

### 3.6. Financial Cost

Cost can be an important factor when deciding on the most appropriate feeding formula for an individual. BTFs are generally cheaper compared with commercial formulas, avoiding the high costs of manufacturing, sterile packaging, processing, complex nutrient fortification and profit margins that are built into commercial products. Of course, the cost of a BTF prepared at home will vary depending on the ingredients and amount needed to prepare the recipe. Unfortunately, although BTFs cost less, this cost is not covered by insurance, giving an advantage to commercial feeds that under certain circumstances, and depending on each country’s policies, are very often covered by health insurance. Furthermore, in a prospective cohort study that aimed to assess healthcare costs associated with CEFs vs BTFs, results showed that BTF-fed patients had significantly fewer hospital admissions, including fewer admissions for respiratory illness and fewer visits to the emergency department, compared with their CEF-fed peers [39]. The study concluded that blenderized diets were associated with decreased healthcare utilization, giving the potential for significant reductions in health care cost. As far as direct cost is concerned, a study conducted at Cincinnati Children’s Hospital estimated that the average daily cost of blended foods was lower compared with that of commercial formulas for the equivalent calories [35]. On the contrary, the other literature suggest that since BTFs are associated with increased tube clogging, connector pieces of syringes and feeding lines and devices may need to be changed more frequently, making the process technically more challenging and potentially more expensive [25,46]. In addition, blending foods is more labor intense and time consuming for the caregiver, which can be considered an indirect cost [29,47].

## 4. Drawbacks Associated with a Blenderized Diet

### 4.1. Microbial Contamination

Concerns have been raised about increased risks of foodborne illness with blenderized feeds, since blended food is not sterile and could consequently result in food poisoning or infections, especially if proper food hygiene and blend storage are not followed [47]. Foodborne illness can result in infection, diarrhea, longer hospital stays and higher mortality rates, especially in critically ill or immunocompromised patients [48,49]. Tube feeds can become contaminated with microorganisms at several time points during preparation, whether in an institution or at home. For instance, the ingredients themselves may be contaminated, the food may not be thoroughly cooked, the person preparing the food may not use suitable hygiene measures, the food may not be properly stored or the blend may not be appropriately handled when put into the feeding system [29,50]. The published literature regarding the microbial content of blenderized feeds is conflicting. In two studies from Iran and the Philippines, where hospital enteral feedings were analyzed for microbial contamination, the results revealed that the microbial quality of the blenderized feeds was not within the acceptable levels for safety [51,52]. In a study conducted in Saudi Arabia, samples of BTFs were collected from three hospitals and were compared with commercial formulas [53]. All samples were analyzed in terms of microbial quality (coliform counts, aerobic plate counts, microorganism growth) and physical characteristics. The results showed that many of the BTF samples had detectable coliform and bacterial counts, whereas no contamination was identified in the commercial formula. On the contrary, in a study from a US hospital, where both CFs and BTFs were analyzed for the growth of aerobic microorganisms, coliforms, Staphylococcus aureus and Escherichia coli, results showed that the total bacterial counts for both feeds were below the acceptable limits and thus, both feeds were found safe for human consumption [54]. On the same line, a study by Baniardalan et al. showed that colony-forming units were significantly higher in CFs compared with BTFs in an intensive care unit patient population at an Iranian teaching hospital [55].

According to the research findings thus far, it seems that methods of enteral feeding preparation and administration, improper food handling and time and temperature violations can influence the microbial load to a greater degree than the type of feed itself [56]. In a study by Milton et al., the authors tried to assess a preparation procedure to minimize the bacterial contamination of BTFs in home settings [56]. The study concluded that established safe food-handling procedures could minimize the bacterial contamination of BTFs and consequently reduce the risk of foodborne infections in home enteral nutrition patients. It is also important to note that no clear data so far show that high microbial loads of BTFs have an effect on patient outcomes. To conclude, emphasis on following good food safety practices such as keeping clean and sanitized kitchen equipment and surfaces, maintaining blenderized formulas at room temperature for no more than two hours and proper refrigeration is of prime importance [57]. Moreover, limiting the hang time of BTFs as much as possible is critical due to the prolonged time the blend is kept at room temperature [57].

### 4.2. Obstruction of the Feeding Tube

Blenderized feeds usually have a higher or inconsistent viscosity than commercial feeds and this can raise the possibility of clogging the feeding tube. In a cross-sectional survey conducted on 1519 home enteral nutrition (HEN) patients (both pediatric and adult) using different types of feeding formulas, the authors assessed, among others, tube clogging complications [58]. Exploring the relationship between formula type and tube clogging, the results showed that BTF users experienced clogs more frequently. Some food-based feeds may be too viscus for administration via gravity and/or an enteral pump and thus, bolus feeding through a gastrostomy tube is the most recommended and common method to provide food-based BTFs [32]. Using a powerful blender and straining the end product will make the mixture thin enough to minimize the possibility of food particles getting stuck in the feeding tube. In order to reduce the viscosity of the feed, dilution might sometimes be required, although overly diluting the feed may necessitate the delivery of a greater volume of feed, extending the time a patient receives EN and, consequently, possibly impacting feed tolerance and quality of life [59,60]. According to the American Society of Parenteral and Enteral Nutrition (ASPEN)’s safe practices for enteral nutrition therapy, using a feeding tube that is at least 14Fr in size or greater can minimize the risk of clogging when on BTFs [57]. Lastly, flushing the tube with water after every feeding is essential to reduce the risk of clogging [61]. It is also worth pointing out that new-generation feeding pumps are enteral feeding pumps and feeding sets capable of delivering thick formulas with improved delivery accuracy [62].

### 4.3. Nutrient Supply

BTFs tend to have an inconsistent and unpredictable micronutrient and macronutrient profile, even when prepared in a hospital environment [30,53]. This inconsistency stems from a number of factors, including the lack of standardized recipes, inconsistent recipe implementation and food variability. Parameters such as harvesting, food preparation and food storage conditions can all affect the nutritional content of a blend [29,53]. For example, the levels of some vitamins in fruits and vegetables can vary if they are stored fresh compared with frozen [63]. Furthermore, as mentioned above, trying to make blends thin enough to pass through a feeding tube may require diluting, and hence, this can limit the provision of nutrients to the patient [60,64]. On the other hand, CEFs have a known and consistent nutritional composition and are often designed for specific diseases and clinical conditions. For instance, semi-elemental or elemental formulas are easier to digest as they contain hydrolyzed macronutrients and can be used in patients with malabsorption [3]. Other disease-specific commercial formulas include renal formulas that are typically volume-restricted with lower levels of electrolytes (phosphorus, potassium, sodium) and pulmonary formulas consisting of a high fat, low carbohydrate ratio, aiming to reduce carbon dioxide production and several other categories [65]. Commercially prepared whole-food formulas have entered the market over the recent years in response to a resurgence of interest in real food for tube feeding, offering convenience and similar benefits to homemade versions without the preparation time. Such feeds can offer an alternative to home-prepared feeds as they can be polymeric, semi-elemental and disease-specific; however, they can be more costly [65]. Table 2 summarizes the advantages and disadvantages associated with blenderized feeds and commercial formulas.

## 5. Conclusions

Enteral nutrition is essential for patients whose oral intake is inadequate for a variety of physiological or medical reasons. It is preferred to parenteral nutrition since it helps maintain gut integrity by maintaining normal digestion and absorption, protecting against gut atrophy. Enteral feeds can be classified into two major categories: commercial enteral formulas and blenderized enteral feeds.

There are advantages and disadvantages associated with both categories of enteral feeds. Over the last years, there has been a growing interest in BTFs since a large portion of the population is shifting towards whole, more natural foods that are less processed. Small-scale, limited emerging research mainly focused on children suggest health benefits associated with BTFs, including improved symptom management, particularly as it relates to gastrointestinal symptoms and a positive impact on gut microbiota. On the contrary, BTFs are linked with an increased risk of microbial contamination and tube clogging.

Further studies, specifically well-designed randomized controlled trials including adult populations that will emphasize the impact of BTFs on various clinical outcomes are warranted. Enteral formula selection can be challenging for clinicians and the use of a patient-centered approach, considering the enteral consumer’s individual needs and current health status when choosing the appropriate enteral feed, is required. Moreover, a multidisciplinary approach to tube feeding is recommended as the etiology of feeding disorders is complex and multifactorial, requiring the expertise of providers across several disciplines. Lastly, clinicians need to be conscientious and educated regarding safe food practices for blended food preparation, as well as the characteristics of commercial formula options available, to help assist patients in selecting the proper feed required for their nutritional needs and clinical condition.

## Figures and Tables

**Table 1:** Studies on the effect of blenderized tube feeds on gastrointestinal tolerance.

Study/Author	Objective	Measured Parameters	Findings/Conclusions
Batsis et al., 2020 [22]	To assess the effect of transitioning children with neurological disorders from commercial formulas to blended diet by gastrostomy tube on gastrointestinal symptoms.	Clinical gastrointestinal symptoms were reviewed at baseline and at 3, 6 and 12 months after blended diet initiation.	Three months after initiation of the blended diet 95% of patients experienced improvements in upper GI symptoms including retching, gagging, emesis, cough and other concerning symptoms for dysphagia or aspiration.
Gallagher et al. [31]	To assess the effect of transitioning chronically ill children from commercial formulas to blenderized feeds via a gastrostomy tube on gastrointestinal symptoms.	Questionnaires to assess caregivers’ perceptions of their child’s GI symptoms, stool frequency and consistency were completed at baseline, and 3 and 6 months.	The percentage of patients vomiting > once a week significantly decreased from 76% to 53%, and the prevalence of gagging and/or retching also decreased considerably from 82% to 47%.
Pentiuk et al., 2011 [35]	To assess the effect of transitioning post-fundoplication children from commercial formulas to a pureed diet by gastrostomy tube on gastrointestinal symptoms.	A telephone survey was conducted asking parents to rate the percentage reduction in gagging and retching since transitioning to the pureed diet.	After transitioning post-fundoplication children from commercial formulas to a pureed diet by gastrostomy tube, 73% had a >50% reduction in gagging and retching.
Schmidt et al., 2019 [36]	The study was carried out on patients recovering from neurological damage, requiring enteral nutrition, who were randomized to receive either blenderized feed or commercial formula over a maximum of 30 days.	The number of defecations and the consistency of each stool according to the Bristol Stool Chart (BSC) were monitored.	Tube feeding with natural-based food was associated with a significant reduction on the number of watery stool evacuations and diarrhea, compared with standard tube feeding.
Hron et al., 2019 [39]	Children receiving blenderized diets versus conventional formulas via feeding tube were assessed in terms of gastrointestinal symptoms.	The Pediatric Gastroesophageal Reflux Disease Symptom and Quality of Life Questionnaire (PGSQ) and PedsQL Gastrointestinal Symptoms Scale (GI-PedsQL) were both used to evaluate gastrointestinal symptoms.	Results showed significantly less nausea, vomiting and abdominal pain in participants who were on blenderized diets.

GI, gastrointestinal.

**Table 2:** Advantages and disadvantages associated with blenderized feeds and commercial formulas.

	Blenderized Feeds	Commercial Formulas
Social and Psychological Implications	Gives a sense of normalcy to patients [20] Patients feel that they are having the same food as the rest of the family	More artificial way of feeding
Anthropometric indices	Some studies have shown positive impacts on improving and maintaining weight and anthropometrics in children patients [22,23,24,25]	Literature has revealed a beneficial effect on anthropometric indices in adult patients/more precise in meeting macronutrient targets [26,27]
Food allergies	BTF’s can allow for careful control over known food allergens as they can be excluded from the feed [24,33,34]	Common food allergens are frequently found in commercially available enteral formulations as they are formulated using ingredients such as eggs, milk, soy, corn and some food additives
Gastrointestinal tolerance	Blenderized feeds are associated with improved gastrointestinal tolerance and a significant reduction in symptoms such as gagging, retching, diarrhea, nausea and vomiting, mainly in pediatric populations [22,31,35,36,39]	Commercial formulas are associated with increased gastrointestinal symptoms due to viscosity characteristics and hormonal responses that affect GI mobility [22,35]
Microbial contamination	The microbial content of blenderized feeds may be higher due to improper food handling, preparation and storage [51,52,53]	Commercial formulas are generally sterile until opened but are not immune to contamination if handled improperly [55].
Gastrointestinal Microbiota	Blenderized diets are associated with increased intestinal bacterial diversity and improved gut microbial composition, mainly in pediatric populations [31,40,41]	Long-term use of commercial formulas may reduce the variety of bacteria in the gut due to being low in fiber and high in ultra-processed refined ingredients [32,44,45]
Obstruction of the tube	BTFs usually have higher or inconsistent viscosity than commercial feeds and this can raise the possibility of clogging the feeding tube [58]	Commercial formulas are generally less likely to cause tube clogging due to their consistent thin viscosity; nevertheless, some types (e.g., high protein, caloric-dense or high fiber) are also associated with a higher risk of tube clogging [3,66]
Nutrient supply	BTFs tend to have an inconsistent and unpredictable micronutrient and macronutrient profile [30,53].	CEFs have a known and consistent nutrition composition and are often designed for specific diseases and clinical conditions [3,65].
Financial cost	BTFs are generally cheaper compared with commercial formulas, avoiding the high costs of manufacturing [35,39]	CEFs are often covered by insurance and are less labor intense and time consuming for the caregiver [29,47].

BTFs, blenderized tube feeds; CEFs, commercial enteral formulas.

## Data Availability

No new data were created or analyzed in this study. Data sharing is not applicable to this article.
